# Immunoglobulin levels of children with bronchiolitis obliterans syndrome after hematopoietic cell transplantation

**DOI:** 10.3389/fmed.2025.1692881

**Published:** 2026-01-05

**Authors:** Yinyan Yue, Kun Yan, Shuai Liu, Suqin Zhang

**Affiliations:** 1 Department of Pediatrics, The First Affiliated Hospital of Zhengzhou University, Zhengzhou, Henan, China; 2 Department of Pediatrics, The Second Affiliated Hospital of Zhengzhou University, Zhengzhou, Henan, China; 3 Department of Pulmonary and Critical Care Medicine, The First Affiliated Hospital of Zhengzhou University, Zhengzhou, Henan, China; 4 Department of Pediatrics, The First Affiliated Hospital of Zhengzhou University, Zhengzhou, Henan, China

**Keywords:** bronchiolitis obliterans syndrome, hematopoietic stem cell transplantation, immunoglobulin, child, deficiency

## Abstract

**Background:**

The development of bronchiolitis obliterans syndrome (BOS) following allogeneic hematopoietic stem cell transplantation (allo-HCT) remains an unresolved clinical problem. However, developing a risk stratification tool for BOS risk is challenging as numerous factors contribute to its development. Moreover, allo-HCT may lead to a decrease in respiratory mucosal surface defense function, resulting in recurrent inflammation and fibrosis. Previous studies have shown that immunoglobulin G (IgG) and IgA levels significantly decrease after lung transplantation and are associated with BOS. Therefore, we hypothesized that immunoglobulin levels of patients may also decrease after allo-HCT, and may even be risk factor for the development of BOS.

**Methods:**

In this retrospective study, a total of 134 patients were enrolled. According to the presence of BOS, these patients were divided into BOS and non-BOS groups. Clinical information and immunoglobulin levels were analyzed between the two groups. Immunoglobulin levels of patients before and after transplantation were compared. Binary logistic regression and Cox regression were used to identify variables with independent prognostic significance.

**Results:**

ABO incompatible, human leukocyte antigen (HLA) mismatch, lung infection within 100 days post-transplantation, cytomegalovirus (CMV) serology positivity, and pre-transplant IgA immunoglobulin deficiency were risk factors for BOS after allo-HCT. Following transplantation, IgA and IgM levels significantly decreased, and many patients had levels below the reference values. In addition, the serum IgA levels prior to transplantation were lower in the BOS group than the non-BOS group. In multivariate models, pre-transplant IgA deficiency was a risk factor for BOS.

**Conclusion:**

Following allo-HCT, IgA and IgM levels decreased, and numerous patients had levels below the reference values. In multivariate models, pre-transplant IgA deficiency was identified as a risk factor for BOS.

## 1 Introduction

Bronchiolitis obliterans (BO) is characterized by persistent inflammation and fibrosis of the small airways, resulting in narrowing and/or complete occlusion of the airway lumen (1); ultimately, this condition may even lead to lung failure. Injury and small airway inflammation can be caused by various factors, such as infection, graft-versus-host immune responses, rejection, chemical inhalation, and autoimmune diseases. Bronchiolitis obliterans syndrome (BOS) is characterized by airflow obstruction and small airway disease features on high-resolution computed tomography in patients undergoing lung transplantation or allogeneic hematopoietic stem cell transplantation (allo-HCT), without the need for pathological confirmation of BO (2).

Respiratory virus infection (3), human leukocyte antigen (HLA) mismatch (4), and positive serology in patients with cytomegalovirus (CMV) (5) are risk factors for the occurrence of BOS. However, the development of a risk stratification tool for BOS is challenging. Currently, there is no specific treatment for BOS; thus, it is important to reduce the incidence of BOS by implementing certain measures.

The respiratory mucosal surface area exceeds 400 m^2^ and contains a large number of potential pathogens (6). The barrier function on the mucosal surface, secretion of antimicrobial peptides and proteins, and body fluids of the immune system, especially immunoglobulin A (IgA) and IgG, can inhibit the pathogenic effects of pathogens. Previous studies have shown that hypogammaglobulinemia was common after organ transplantation and associated with surgical infection complications (7). Following lung transplantation, IgG and IgA levels significantly decreased and were associated with BOS (8). Given the incidence of immunoglobulin deficiency after organ transplantation and the role of low immunoglobulin in the pathogenesis of BOS, we hypothesized that immunoglobulin deficiency, particularly IgA deficiency, is an important driving factor for BOS after HCT. Moreover, further understanding the role of immunoglobulin in BOS can promote the development of targeted immunotherapy, thereby delaying or preventing the occurrence and development of BOS.

## 2 Materials and methods

### 2.1 Study design

In this retrospective study, a total of 134 patients were enrolled. The inclusion criteria were: (1) the guardians of the patients agreed to receive allo-HCT at the First Affiliated Hospital Zhengzhou University and provided written informed consent (transplantation period: January 1, 2019—December 31, 2022); (2) patient age ranging: 0–16 years; (3) clear diagnosis of the primary disease and meeting the indications of allo-HCT; (4) complete clinical data; and (5) survival for at least 100 days after allo-HCT. The exclusion criteria were: (1) refusal to follow up; and (2) diagnosis of pulmonary disease or dysfunction (e.g., asthma, idiopathic pulmonary fibrosis, post-infection BO, cystic fibrosis, pulmonary hypertension) prior to transplantation. This study was approved by the First Affiliated Hospital of Zhengzhou University.

### 2.2 Definition of BOS after allo-HCT

Following allo-HCT, the diagnosis of BOS was based on the following diagnostic criteria (9) of the pulmonary function test in the revised NIH standard (10): (1) forced expiratory volume in 1 s (FEV1) < 75% of predicted or a decrease of the FEV1 by 10% in comparison to the pretransplant value; (2) FEV1/forced vital capacity (FVC) < 70%; (3) residual volume (RV) or RV/total lung capacity (TLC) > 120% of predicted; and (4) evidence of air trapping on high-resolution computed tomography (HRCT).

### 2.3 Measurements of immunoglobulin levels

The levels of IgA, IgG, IgM, C3 and C4 were collected before allo-HCT related immunosuppression or myeloablative treatment and after allo-HCT. The normal reference values for IgG, IgA, and IgM are > 7, > 1, > 0.4 g/L, respectively, as previously reported (11). Immunoglobulin deficiency is defined as serum levels below the lower limit of the predicted normal value.

### 2.4 Clinical information

Clinical parameters data were collected, including age at onset, recipient sex, recipient age, donor sex, underlying disease, time from diagnosis to transplant, months, donor type, ABO blood type, HLA, use of busulfan, antithymocyte globulin (ATG), mesenchymal stem cells (MSCs), pulmonary infection within 100 days prior to transplant, pulmonary infection within 100 days after transplantation, and infection with Epstein-Barr virus (EBV) or CMV following transplantation.

### 2.5 Statistical analysis

All data were analyzed by SPSS version 27.0 software. The Kolmogorov-Smirnov method was used to analyze the normality of continuous variables. For normally distributed data, the independent *t*-test was used to compare the differences between continuous variables; for non-normally distributed data, the Mann–Whitney U-test was used. Categorical data analysis and comparison of the two groups were conducted using the chi-squared or Fisher’s exact tests. Paired *t*-tests were performed to compare data obtained before and after allo-HCT in patients. The results were presented as the mean ± standard deviation for numerical variables and number (percent) for categorical data. Binary logistic regression was used to analyze risk factors for BOS. Cox regression was used to identify variables with independent prognostic significance. *P* < 0.05 indicate statistically significant difference.

## 3 Results

### 3.1 Clinical and transplant features of patients

A total of 134 patients who underwent allo-HCT at the First Affiliated Hospital Zhengzhou University from January 1, 2019 to December 31, 2022 were enrolled in this study. The clinical and transplant characteristics are shown in Table 1. Pulmonary function tests of BOS group after transplantation were as follows: FEV1% (51.89 ± 15.892)%, FEV1/FVC (62.76 ± 8.179)%, maximum mid-term expiratory flow rate (28.26 ± 10.269) L/s, FEF75% (23.76 ± 10.181)%. And air trapping signs can be seen on lung CT of children in the BOS group. There were no differences in the age at onset and the age of recipients between the BOS and non-BOS group. Moreover, there were no differences between the two groups in the sex ratio of donors and recipients, the usage of busulfan, ATG, and MSCs. Patients had higher ratio of ABO incompatibility (79% vs. 48%, *p* = 0.046), HLA mismatch (86% vs. 52%, *p* = 0.022), and co-existence of rejection and infection after-transplantation in the BOS group than the non-BOS group. Additionally, more patients in the BOS group had lung infection within 100 days after-transplantation (79% vs. 51%, *p* = 0.049) and CMV infection after-transplantation (50% vs. 20%, *p* = 0.012) compared with the non-BOS group. The probability of developing extrapulmonary organ rejection was higher for patients with BOS than for those without BOS (79% vs. 42%, *p* = 0.012). However, since chronic graft-versus-host disease (GVHD) was included as one of the criteria in defining BOS in this study, it was not included for the subsequent analysis of BOS risk factors.

**TABLE 1:** Clinical characteristics of patients with and without BOS.

Clinical variable	BOS (*n* = 14)	Non-BOS (*n* = 120)	*p-*value
Onset age (month)	70.9 ± 45.083	92.8 ± 48.282	0.108
Recipient age (month)	86.7 ± 52.60	107.9 ± 47.02	0.117
Recipient sex, male	8(57)	82(68)	0.548
Donor sex, male	8(57)	79(66)	0.561
**Underlying disease**			
AA	5(36)	55(46)	0.511
ALL	4(28.5)	19(16)	
AML	4(28.5)	27(22)	
Other	1(7)	19(16)	
Time from diagnosis to transplant, months	15.9 ± 24.95	15.2 ± 24.78	0.92
**Donor type**			
Sibling/unrelated	5(36)/9(64)	70(58)/50(42)	0.154
**ABO**			
Compatibility/incompatibility	3(21)/11(79)	62(52)/58(48)	0.046
**HLA**			
Full match/mismatch	2(14)/12(86)	57(48)/63(52)	0.022
Busulfan-based/other	13(93)/1(7)	91(76)/29(24)	0.191
ATG/other	12(86)/2(14)	97(81)/23(19)	0.742
MSCs/other	11(79)/3(21)	84(70)/36(30)	0.559
Acute GVHD/other	8(57)/6(43)	45(38)/75(62)	0.247
Extrapulmonary organ rejection/other	11(79)/3(21)	51(42)/69(58)	0.012
Pulmonary infection within 100 days pre-transplantation	8(57)/6(43)	46(38)/74(62)	0.249
Pulmonary infection within 100 days after-transplantation	11(79)/3(21)	61(51)/59(49)	0.049
Infection within 100 days after-transplantation	11(79)/3(21)	69(58)/51(42)	0.128
EB virus infection after transplantation	0(0)/14(100)	4(3)/116(97)	0.488
CMV virus infection after transplantation	7(50)/7(50)	24(20)/96(80)	0.012
**Rejected organ**			
Lung	2(14)	0(0)	< 0.001
Skin	4(29)	31(26)	
Liver	0(0)	2(2)	
Intestine	0(0)	7(6)	
Two or more organs	7(50)	11(9)	
None	1(7)	69(57)	
Co-existence of rejection and infection after-transplantation	6(43)	8(7)	< 0.001

AA, aplastic anemia; AML, acute myeloid leukemia; ALL, acute lymphoblastic leukemia; ABO, ABO blood group; ATG, anti-human thymoglobulin; BOS, bronchiolitis obliterans syndrome; CMV virus, Cytomegalovirus; EBV, Epstein-Barr virus; GVHD, graft-versus-host disease; HLA, human leukocyte antigen; MSCs, mesenchymal stem cells. Data are expressed as median ± standard deviation or numbers (%).

### 3.2 Lower immunoglobulin levels after allo-HCT

**FIGURE 1:**
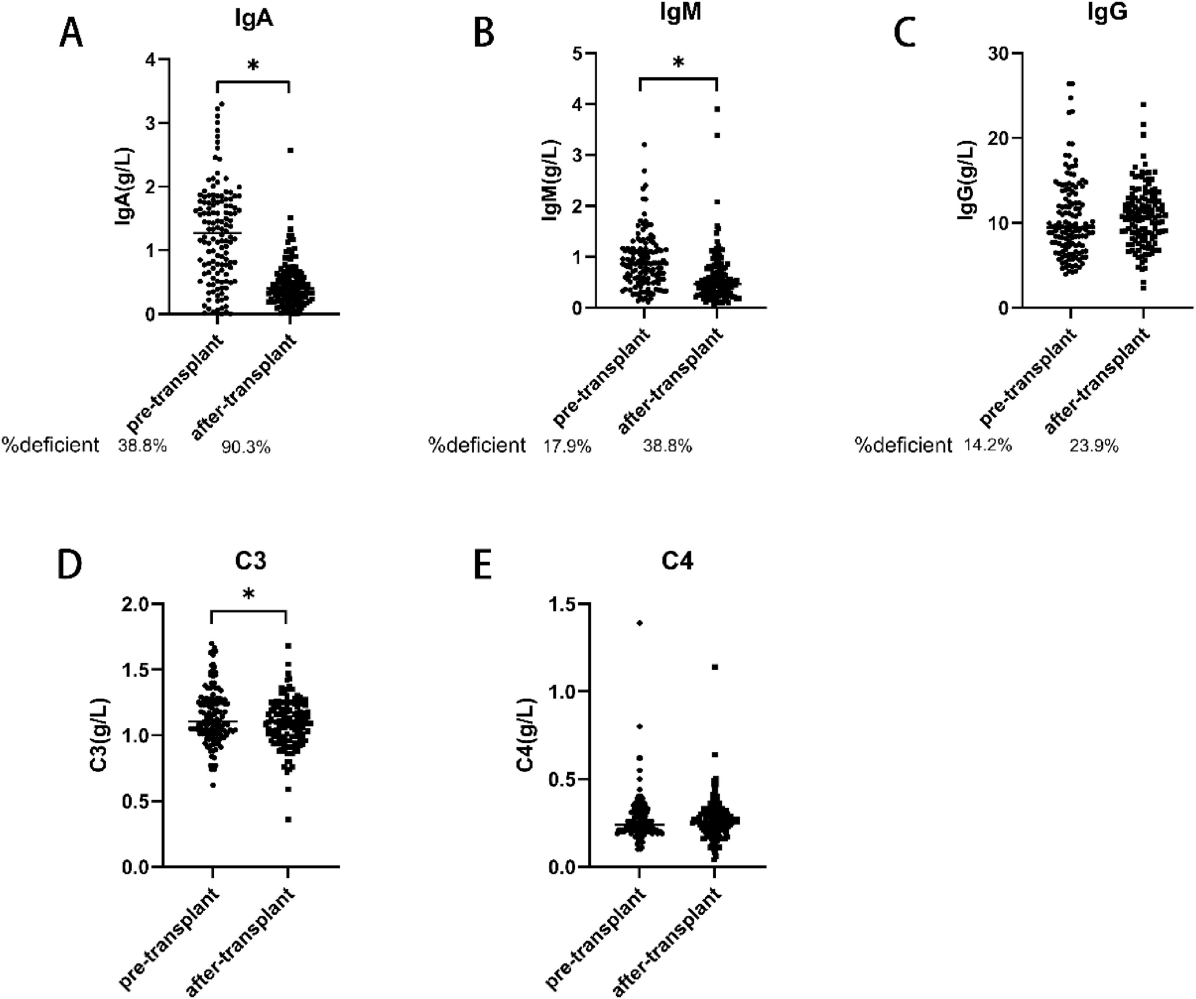
Immunoglobulin levels before and after transplantation. The levels of IgA and IgM and C3 were significantly lower post-transplantation than before transplantation (**A,B,D**). There were no significant differences in the levels of IgG and C4 before and after transplantation (**C,E**). IgA, immunoglobulin A; IgG, immunoglobulin G; IgM, immunoglobulin M.*p < 0.05 vs. pre-transplant.

However, the immunoglobulin levels in children before and after allo-HCT remain unknown. Therefore, in this study, we analyzed the immunoglobulin levels of the children before and after allo-HCT. The results showed that 38.3%, 17.9, and 14.2% of the patients were IgA-deficient (1.25 ± 0.762 g/liter), IgM-deficient (0.90 ± 0.531 g/L), and IgG-deficient (10.27 ± 4.206 g/liter) (Figure 1), respectively, before allo-HCT related immunosuppression or myeloablative treatment. Only modest correlations were observed between IgM and IgA levels, IgG and IgA levels, and IgG and IgM levels (*r* = 0.229, *p* = 0.008; *r* = 0.326, *p* < 0.001; *r* = 0.247, *p* = 0.004). The levels of IgA (0.47 ± 0.362 vs. 1.25 ± 0.762, *p* < 0.001), IgM (0.58 ± 0.518 vs 0.90 ± 0.531, *p* < 0.001) and C3 (1.09 ± 0.184 vs. 1.15 ± 0.210, *p* = 0.017) after-transplant were significantly lower after transplantation than before transplantation. However, there was no significant change in the levels of IgG and C4 after allo-HCT (Figure 1).

### 3.3 Immunoglobulin levels before and after allo-HCT in BOS and non-BOS groups

**FIGURE 2:**
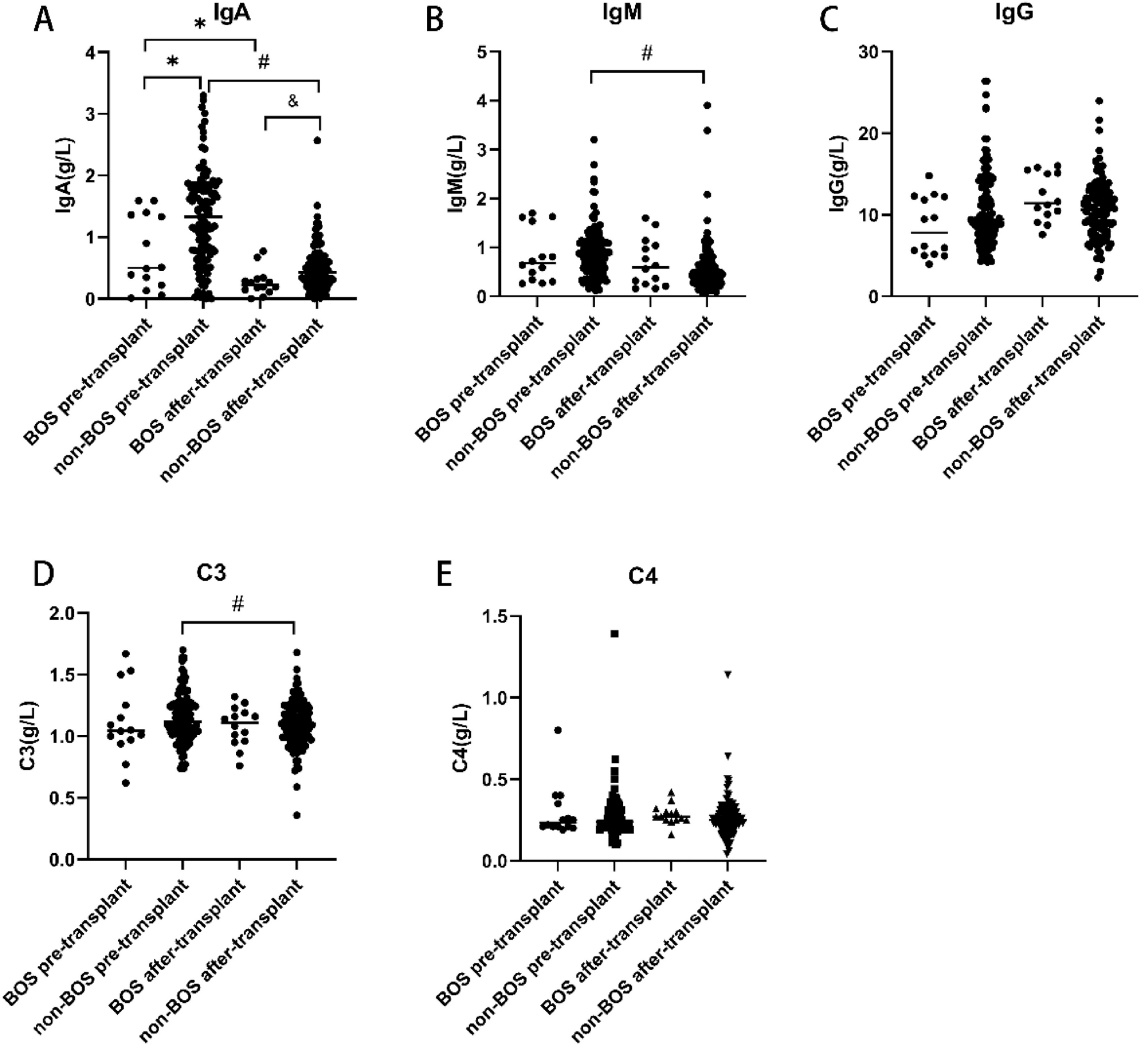
Immunoglobulin levels before and after transplantation in the BOS and non-BOS group. The levels of IgA were significantly lower in the BOS group than the non-BOS group (before and following transplantation) **(A)**. In the non-BOS group, the levels of IgM and C3 were markedly lower after transplantation than before transplantation **(B,D)**. There were no differences in IgG and C4 levels among groups **(C,E)**. BOS, bronchiolitis obliterans syndrome; IgA, immunoglobulin A; IgG, immunoglobulin G; IgM, immunoglobulin M. *p < 0.05 vs. BOS pre-transplant, ^#^*p* < 0.05 vs. non-BOS pre-transplant, ^&^*p* < 0.05 vs. BOS after-transplant.

Both the BOS group (0.27 ± 0.216 g/L vs. 0.76 ± 0.620 g/L, *p* = 0.013) and non-BOS group had lower IgA levels after transplantation than before transplantation (0.49 ± 0.367 g/L vs. 1.31 ± 0.751 g/L, *p* < 0.001). In addition, the IgA levels before (*p* = 0.012) and after (*p* = 0.027) transplantation were significantly lower in the BOS group than the non-BOS group. The levels of IgM (0.57 ± 0.522 g/L vs. 0.91 ± 0.531 g/L, *p* < 0.001) and C3 (1.09 ± 0.187 vs. 1.15 ± 0.199, *p* = 0.011) after transplantation were also significantly lower in the non-BOS group than the non-BOS group. In the BOS group, the IgM levels were lower after transplantation than before transplantation; however, the difference was not statistically significant. Of note, there were no difference in IgG and C4 levels between the groups (Figure 2).

### 3.4 Serum IgA levels before transplantation were associated with prevalence and incidence of BOS

The results of the multivariate analysis of IgA and IgM levels were summarized in Tables 2, 3. Recipient age was identified as independent predictors of low post-transplantation serum IgA levels (Table 2). Recipient age was identified as an independent predictor of low post-transplantation serum IgM levels (Table 3).

**TABLE 2:** Factors significantly associated with serum IgA level after transplantation.

Variables	β (95% CI)	*p*
**Univariate analysis**		
Onset age (month)	0.002(0.000, 0.003)	0.009
Recipient age (month)	0.002(0.001, 0.004)	< 0.001
Time from diagnosis to transplant, months	0.002(0.000, 0.005)	0.060
Presence of BOS	−0.225(−0.424, −0.027)	0.027
**Multivariate analysis**		
Recipient age (month)	0.002(0.001, 0.003)	0.002

CI, confidence interval.

**TABLE 3:** Factors significantly associated with serum IgM level after transplantation.

Variables	β (95% CI)	*p*
**Univariate analysis**		
Onset age (month)	0.002(0.001, 0.004)	0.051
Recipient age (month)	0.003(0.001, 0.004)	0.004
**Multivariate analysis**		
Recipient age (month)	0.004(0.001, 0.008)	0.020

CI, confidence interval.

Predictors for BOS are summarized in Table 4. In the univariate analysis, ABO, HLA, pulmonary infection within 100 days after transplantation, CMV infection after transplantation, and IgA levels before and after transplantation were identified predictors. In a multivariate model, lower serum IgA levels before transplantation (hazard ratio,0.259; 95%confifidence interval, 0.081−0.824; *p* = 0.022) were associated with a higher incidence of BOS.

**TABLE 4:** Factors significantly associated with bronchiolitis obliterans syndrome.

Variables	HR (95% CI)	*p*
**Univariate analysis**		
ABO (incompatibility)	3.92(1.041, 14.759)	0.043
HLA (mismatch)	5.429(1.165, 25.303)	0.031
Pulmonary infection within 100 days after-transplantation	3.546(0.942, 13.353)	0.061
CMV serology positivity	4.00(1.280, 12.496)	0.017
IgA before transplantation	0.307(0.118, 0.798)	0.015
IgA after transplantation	0.038(0.002, 0.621)	0.022
**Multivariate analysis**		
IgA before transplantation	0.259(0.081, 0.824)	0.022

CI, confidence interval.

## 4 Discussion

The present results showed a significant decrease in serum IgA and IgM levels after allo-HCT, with many patients having levels below the reference values. In addition, the serum IgA levels were lower in the BOS group than the non-BOS group prior to transplantation. Moreover, pre-transplantation IgA deficiency was a risk factor for BOS onset in multivariate modeling.

Allo-HCT provides the best chance for cure in many patients with malignant and non-malignant hematologic disorder, such as acute myeloid leukemia (AML), acute lymphoblastic leukemia (ALL), chronic myeloid leukemia (CML), myelodysplastic syndrome, aplastic anemia (AA), Mediterranean anemia, non-Hodgkin’s lymphoma (12), certain solid tumors (13), autoinflammatory disorders (14), and other diseases. The development of BOS after allo-HCT remains an unresolved clinical issue, as once it occurs, treatment becomes difficult (15).

BOS is defined by a progressive obstructive ventilatory impairment resulting from obliterative bronchiolitis (16). Its pathogenesis following allo-HCT involves chronic graft-versus-host disease (GVHD), which arises from a breakdown in central tolerance and dysregulated B-cell activity with autoantibody production (17). Azithromycin represents the most effective initial therapy for BOS, although treatment response rates remain modest, ranging from 29 to 50% (18). Montelukast, an oral agent targeting cysteinyl leukotriene (CysLT) receptors in the airways, has been associated with neuropsychiatric adverse effects—including depression, anxiety, sleep disturbances, agitation, and paresthesia—particularly in patients with a pre-existing psychiatric history (19), regardless of age. Second-line therapeutic options include extracorporeal photopheresis (ECP) and total lymphoid irradiation (TLI) (20). However, ECP is limited by its high cost, limited availability, and logistical challenges. While TLI has been shown to attenuate the decline in FEV1 among BOS patients, the supporting evidence is derived primarily from small observational studies (21). For carefully selected patients who fail first- and second-line therapies, lung re-transplantation represents the principal therapeutic alternative in cases of refractory allograft dysfunction (22). Thus, it is important to prevent its occurrence by implementing appropriate measures. However, the development of a risk stratification tool for BOS is challenging. Peripheral blood as a stem cell source (10), busulfan-based conditioning, unrelated donors, female donors (23), respiratory viral infections (3), HLA mismatch (4), CMV serology positivity (5), etc. are risk factors for the development of BOS. Our results also showed that ABO incompatibility, HLA mismatch, pulmonary infection within 100 days after-transplantation, CMV serology positivity, and pre-transplant IgA deficiency were risk factors for BOS following allo-HCT. Since chronic GVHD was included in the diagnostic criteria, we did not analyze its role as a risk factor for BOS. Therefore, the discovery of additional factors that can exert protective effects on patients receiving allo-HCT plays an important role in preventing the occurrence of BOS.

Immunoglobulin is a heterodimeric protein composed of two heavy chains and two light chains. According to the constant domain of the immunoglobulin heavy chain, it is divided into five categories, namely IgM, IgG, IgA, IgD, and IgE isotypes (24). Immunoglobulin has two main functions of immunoglobulin. Firstly, as a cell surface receptor, it binds to antigens, transmits cell signals, and activates cells. Secondly, as a soluble effector molecule, it independently binds and neutralizes antigens within a certain distance (25). IgM antibodies participate in primary immune responses and are often used clinically to diagnose acute pathogen infections. IgG is a major isotype found in the body that directly contributes to immune responses, including neutralizing toxins and viruses. The levels of IgA in serum are often higher than those of IgM, but markedly lower than the levels of IgG (26). However, IgA is the most abundant immunoglobulin in the mucosa, playing a key role in host defense against inhaled and ingested pathogens and mucosal immune regulation (27). IgA deficiency is physiologic in infants (28). Secretions, such as saliva and breast milk, contain high levels of IgA; IgA in breast milk can provide protection for the intestines of infants. Investigations have shown that a lack of IgA in the bloodstream suggests a similar deficiency in local mucosal IgA, and IgA in the bloodstream plays an important role in lung mucosal defense (11). Previous studies have demonstrated that humoral immunity plays an important role in the evolution of BOS (29) in patients with BOS following lung transplantation (1). Furthermore, IgA (11) and IgG deficiencies after lung transplantation (30, 31) were identified as risk factors for BOS. Our results showed that the levels of IgA and IgM were significantly lower after transplantation than before transplantation. In addition, the serum IgA levels prior to transplantation were lower in the BOS group than the non-BOS group. These results indicate that airway mucosal immunity and acute infection immune response are reduced after allo-HCT, and initial airway mucosal immunity plays an important role in the occurrence and development of BOS. Therefore, except for the recipient age, we hypothesize that the occurrence of BOS can be reduced by improving the immune function of patients.

The present study had certain limitations. This was a single-center retrospective investigation involving a short study period and 14 patients diagnosed with BOS. Research including longer study periods and more patients is warranted to further examine the levels of immunoglobulin in this setting. Furthermore, it is necessary to increase the number of time points for the detection of immunoglobulin levels after transplantation to better understand the changes.

## 5 Conclusion

Following allo-HCT, IgA and IgM levels decreased, and numerous patients had levels below the reference values. In multivariate models, pre-transplant IgA deficiency was identified as a potential risk factor for BOS.

## Data Availability

The raw data supporting the conclusions of this article will be made available by the authors, without undue reservation.
